# Genome-wide association studies reveal novel loci for resistance to groundnut rosette disease in the African core groundnut collection

**DOI:** 10.1007/s00122-023-04259-4

**Published:** 2023-03-10

**Authors:** Esther Achola, Peter Wasswa, Daniel Fonceka, Josh Paul Clevenger, Prasad Bajaj, Peggy Ozias-Akins, Jean-François Rami, Carl Michael Deom, David A. Hoisington, Richard Edema, Damaris Achieng Odeny, David Kalule Okello

**Affiliations:** 1 Department of Agricultural Production, College of Agricultural and Environmental Sciences, Makerere University, P.O. Box 7062, Kampala, Uganda; 2 Regional Study Center for the Improvement of Drought Adaptation, Senegalese Institute for Agricultural Research, BP 3320, Thiès, Senegal; 3 UMR AGAP, CIRAD, 34398 Montpellier, France; 4 UMR AGAP, CIRAD, BP 3320, Thies, Senegal; 5 HudsonAlpha Institute for Biotechnology, Huntsville, AL 35806 USA; 6 International Crops Research Institute for the Semi-Arid Tropics, Patancheru, Telangana 502324 India; 7 Center for Applied Genetic Technologies, University of Georgia, Tifton, GA 31793 USA; 8 CIRAD, INRAE, AGAP, Univ Montpellier, Institut Agro, 34398 Montpellier, France; 9 Department of Pathology, The University of Georgia, Athens, GA 30602 USA; 10 Feed the Future Innovation Lab for Peanut, University of Georgia, Athens, GA 30602 USA; 11 Makerere University Regional Center for Crop Improvement Kampala, P.O. Box 7062, Kampala, Uganda; 12 International Crops Research Institute for the Semi-Arid Tropics, PO Box, Nairobi, 39063-00623 Kenya; 13 National Semi-Arid Resources Research Institute-Serere, P.O. Box 56, Kampala, Uganda

## Abstract

**Key message:**

We identified markers associated with GRD resistance after screening an Africa-wide core collection across three seasons in Uganda

**Abstract:**

Groundnut is cultivated in several African countries where it is a major source of food, feed and income. One of the major constraints to groundnut production in Africa is groundnut rosette disease (GRD), which is caused by a complex of three agents: groundnut rosette assistor luteovirus, groundnut rosette umbravirus and its satellite RNA. Despite several years of breeding for GRD resistance, the genetics of the disease is not fully understood. The objective of the current study was to use the African core collection to establish the level of genetic variation in their response to GRD, and to map genomic regions responsible for the observed resistance. The African groundnut core genotypes were screened across two GRD hotspot locations in Uganda (Nakabango and Serere) for 3 seasons. The Area Under Disease Progress Curve combined with 7523 high quality SNPs were analyzed to establish marker-trait associations (MTAs). Genome-Wide Association Studies based on Enriched Compressed Mixed Linear Model detected 32 MTAs at Nakabango: 21 on chromosome A04, 10 on B04 and 1 on B08. Two of the significant markers were localised on the exons of a putative TIR-NBS-LRR disease resistance gene on chromosome A04. Our results suggest the likely involvement of major genes in the resistance to GRD but will need to be further validated with more comprehensive phenotypic and genotypic datasets. The markers identified in the current study will be developed into routine assays and validated for future genomics-assisted selection for GRD resistance in groundnut.

**Supplementary Information:**

The online version contains supplementary material available at 10.1007/s00122-023-04259-4.

## Introduction

Cultivated groundnut (*Arachis hypogaea* L.) (2*n* = 4*x* = 40), also known as peanut, is an important cash and food crop worldwide (Okello et al. 2013; Janila et al. 2016). It is cultivated in more than 100 countries with an estimated average annual world production of 49 million tonnes (FAOSTAT 2019). Asia is the leading continent in groundnut production (~56%) followed by Africa (~34%) (FAOSTAT 2019). The kernels are a rich source of dietary protein (Arya et al. 2016; Toomer 2017), healthy fats (Mora-Escobedo et al. 2015), essential vitamins (King et al. 2008; Arya et al. 2016) and micronutrients (Mienie et al. 2013; Kurapati et al. 2021), making it an important ingredient in the formulation of ready to use therapeutic foods (RUTF) for target populations in Africa and Asia (Nabuuma et al. 2013; Wagh and Deore 2015; Schoonees et al. 2019). Groundnut haulm and seed cake are preferred sources of fodder and feed (Desmae et al. 2019; Ahmed et al. 2021). Other industrial uses include making soaps, detergents, paints, cosmetics, candles and lubricants (Janila et al. 2016).

Despite being the second most important legume crop after common bean (*Phaseolus vulgaris*) in many sub-Saharan African (SSA) countries, groundnut productivity is extremely low, owing to various biotic and abiotic challenges. One of the most important foliar diseases in SSA is Groundnut Rosette Disease (GRD), which is endemic to SSA and was first reported in Tanzania in 1907 (Naidu et al. 1999). GRD has since spread to several countries in SSA and its offshore islands leading to losses of up to 100% in pod yield, especially if the symptoms occur before flowering (Okello et al. 2010, 2014). A complex of three agents that function in a synergistic manner cause GRD; groundnut rosette assistor luteovirus (GRAV); groundnut rosette umbravirus (GRV) and its satellite RNA (satRNA) (Naidu et al. 1999; Deom et al. 2000). The satellite RNA depends on GRV for its replication and on GRAV for its encapsidation (Taliansky et al. 2000). Aphids (*Aphis craccivora* Koch) are the principal transmission vectors for the GRD agents (Lynch 1990).

The presence of all the three disease agents results in severely stunted and bushy plants with reduced leaf size and shortened internodes (Waliyar et al. 2007; Nigam et al. 2012). Sole infection from GRAV or GRV agents alone result in either no symptoms or in a mild transient mottle or yellowing in groundnut foliage (Waliyar et al. 2007). The main cause of GRD damage is the GRV-satRNA (Murant and Kumar 1990; Taliansky et al. 2000), which is responsible for symptoms ranging from green (Okello et al. 2014, 2017; Mabele et al. 2021), chlorotic/yellow (Okello et al. 2017; Mabele et al. 2021) and mosaic rosette (Waliyar et al. 2007; Mukoye et al. 2020). Although there is evidence suggesting that different forms of satRNA from different regions of the world may be responsible for different symptoms (Murant and Kumar 1990; Mukoye et al. 2020), the studies are not comprehensive enough to be conclusive on the specific satRNA forms causing yellow, green or mosaic symptoms. Disease scoring has, therefore, been done according to the number of plants showing at least one of the GRD symptoms rather than by the types of symptoms observed (Reddy 1991; Waliyar et al. 2007; Mugisa et al. 2016; Mukoye et al. 2020).

The most effective and practical solution for groundnut farmers is to grow GRD resistant varieties (Nigam et al. 2012). However, the complexity of the viral agents and the involvement of a transmission vector have made successful breeding for complete resistance difficult. Previous reports suggest that resistance could be specific to the various agents (Waliyar et al. 2007) or to the vector (Minja et al. 1999). Furthermore, the genetics of resistance to the disease agents or the vector are not clearly understood (Bock et al. 1990; Olorunju 1992; Herselman et al. 2004; Usman et al. 2015; Athanas 2015; Nalugo et al. 2016). Although efficient development of resistant varieties in other crops with similar complex diseases has been possible through the use of molecular markers (Awata et al. 2021), groundnut breeding programs in Africa are largely conventional. The few reported molecular studies deployed include Amplified Fragment-Length Polymorphisms (AFLPs) for resistance to aphids (Herselman et al. 2004) and Simple Sequence Repeat (SSR) markers for GRD resistance (Pandey et al. 2014; Athanas 2015). However, the reported associated markers were not validated and, therefore, are not used routinely in any of the breeding programs in SSA.

Recent developments in groundnut genomics (Bertioli et al. 2016, 2019; Pandey et al. 2017; Clevenger et al. 2018; Korani et al. 2019) provide great opportunities for enhanced utilization of state-of-the-art molecular markers in breeding programs in SSA and elsewhere. Linkage disequilibrium (LD) or association mapping has rapidly become a useful method in elucidating the molecular basis underlying phenotypic variation (Alqudah et al. 2020). Genome Wide Association Studies (GWAS) have been used to identify molecular markers and Quantitative Trait Loci (QTLs) associated with economically important traits in groundnut (Wang et al. 2019; Zhang et al. 2020, 2021; Otyama et al. 2022). The only reported GWAS study that involved GRD resistance in groundnut (Pandey et al. 2014) used germplasm from the ‘reference set’, majority of which are not part of the SSA breeding programs. In this study, we performed a GWAS for GRD resistance using 213 genotypes selected from the African core collection. Our aim was to exploit the natural variation present in this representative set of genotypes to identify novel sources of resistance to GRD, associated molecular markers and putative genes.

## Materials and methods

### Plant material

**Fig. 1:**
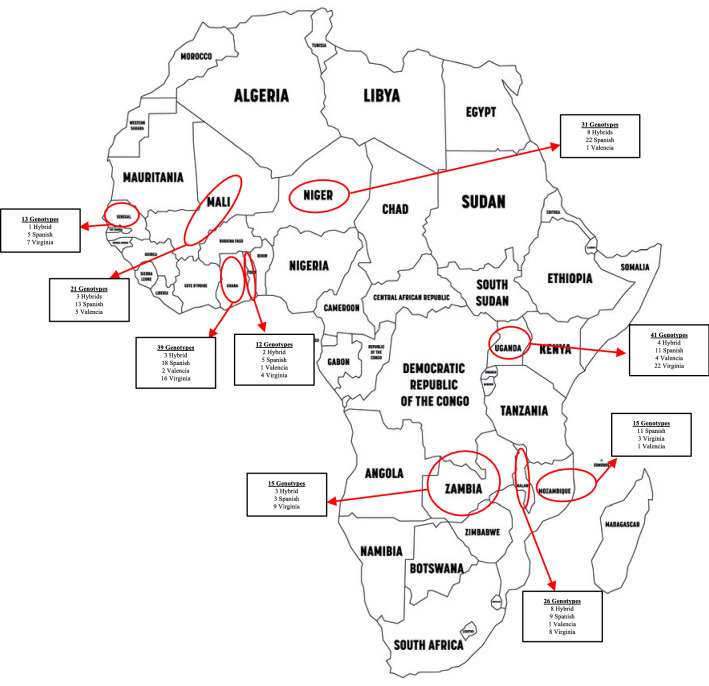
A map of Africa showing countries from which genotypes for core collection were obtained and their market classes

Two-hundred and thirteen (213) breeding lines from nine African countries that were part of the African core collection were used in this study (Supplementary Table 1; Fig. 1). The African core collection was constructed from a nucleus of 116 non-redundant breeders-preferred genotypes and expanded to 300 genotypes using genotyping data and the core hunter software (De Beukelaer et al. 2018). The 213 genotypes of the subspecies fastigiata (32 “hybrid” (combinations between botanical types), 97 Spanish, 10 Valencia) and the hypogaea subspecies (74 Virginia) used in this study were selected based on availability of seed for multi-location trials. Each trial contained a maximum of 200 genotypes per season depending on seed availability.

### Field screening and evaluation for disease resistance

Field evaluation was done in Eastern Uganda at two GRD hotspot locations, Serere and Nakabango (Okello et al. 2010). Serere is located 33A°26′43.943″ E and 1A°31′58.580″ N at 1126 m above sea level while Nakabango is located 33^o^12′47.588″ E and 0^o^31′26.762″ N at 1169 m above sea level. The 200 lines were planted in two 1-m row plots at a spacing of 15 cm within rows and 45 cm between rows in a 10 × 20 lattice design. The trial was planted in two replicates across the two locations in three seasons (2020A, 2020B and 2021B). Genotypes Ug-43_Oug-RED_BEAUTY_UG and Gh2-54_GhaII-NUMEX_03 were used as susceptible checks, while Ug-41_Oug-DOK_1_RED_UG and Ug-194_Oug-ICGV_90099 were used as resistant checks (Supplementary Table 1).

GRD incidence was recorded based on the intensity and presence of any one of the symptoms recorded in literature (Waliyar et al. 2007; Okello et al. 2014, 2017; Mukoye et al. 2020; Mabele et al. 2021). Percentage GRD incidence (Waliyar et al. 2007) was recorded at 30, 60 and 90 days after planting (DAP). Percentage disease incidence (PDI) was calculated as:

$${\text{GRD PDI }}\left( \% \right) = \left( {\frac{{{\text{Number of plants showing rosette symptoms}}}}{{{\text{Plant stand count at a given crop stage}}}}} \right) \times 100$$

The PDI data at 30, 60 and 90 days were used to calculate the Area Under Disease Progress Curve (AUDPC) using the formula:

$${\text{AUDPC}} = \mathop \sum \limits_{i = 1}^{n - 1} \left( {\frac{{y_{i} + y_{i + 1} }}{2}} \right) \left( {t_{{i + 1^{ - } }} t_{i} } \right)$$

where *y*_*i*_ is the PDI at the *i*th observation; *t*_*i*_ is time (in days) at the *i*th observation and *n* is the total number of observations (Simko and Piepho 2012).

### Statistical analysis of phenotypic data

Best linear unbiased predictions (BLUPs) and thereafter variance components within environments were estimated in the lme4 package (Bates et al. 2015) in R (R core team 2021) by manipulating the REstricted Maximum Likelihood (REML) method using the model:

$$Y_{{{\text{ijk}}}} = \mu + G_{i} + R_{j } + R/B_{{{\text{jk}} }} + \varepsilon_{{{\text{ijk}}}}$$

where *Y*_ijk_ is the *k*th observation for the *i*th genotype; *µ* is the overall mean; *G*_*i*_ is the Genotype effect, *Rj* is the replication effect while *R/B*_jk_ is the effect of blocks nested in replicates, respectively; *ε*_ijk_ is the error term associated with *Y*_ijk_*.*

BLUP variance components estimated within environments were appropriated to calculate Broad-sense heritability (*H*^*2*^* bs*) for GRD using the formula:

$$H^{2 } bs = \frac{{\sigma^{2} g}}{{\left( {\sigma^{2 } g + {\raise0.7ex\hbox{${\sigma^{2 } e}$} \!\mathord{\left/ {\vphantom {{\sigma^{2 } e} {nr}}}\right.\kern-0pt} \!\lower0.7ex\hbox{${nr}$}}} \right)}}$$

where *σ*^2^*g* is the genetic variance component and *σ*^2^*e* is the residual (error) component and nr is the number of replications.

BLUPs were further used to generate frequency distribution curves and in GWAS.

### DNA isolation, genotyping and SNP calling

Three seeds per genotype were planted per pot in the screen house at the Regional Center for Drought Adaptation Improvement (CERAAS) in Senegal West Africa. Thinning was done to retain one plant per genotype. Twenty mg of oven-dried young leaves from a single plant were collected 21 days after planting. DNA was isolated using the MATAB protocol (Gawel and Jarret 1991) and purified using the Zymo DNA purification Kit (ZYMO Research USA). A final concentration of 100 ng/µl was obtained for genotyping.

Genotyping was done using the Thermofisher SNP array Axiom *Arachis*2 with 48 K SNPs (Clevenger et al. 2018; Korani et al. 2019). SNP data were extracted from raw files and filtered using Axiom Arachis Suite Version 4.0.3 from Thermofisher scientific (https://www.thermofisher.com/fr/fr/home/life-science/microarray-analysis/microarray-analysis-instruments-software-services/microarray-analysis-software/axiom-analysis-suite.html). The raw SNPs were filtered at a call rate > 0.95 and minor allele frequency > 0.05. The distribution of the final filtered high-quality SNPs was plotted across the chromosomes using CMplot (Yin et al. 2021).

### Genetic diversity, population structure and linkage disequilibrium (LD)

Filtered SNPs were used to draw a Neighbor-Joining dendrogram in TASSEL 5.2.67 (Bradbury et al. 2007). Principal Component Analysis (PCA) was done in the SNP & Variation Suite (SVS version 8.9.0). Ten principal components (PCs) and the additive model were used to generate Eigen values. The first three principal components of the variation were plotted and visualized in R software using the scatterplot3d 0.3–41 package (Ligges and Machler 2003). The Discriminant Analysis of Principal Components (DAPC) was done using the adegenet v. 2.1.5 package in R software by retaining fifty principal components and clustering the genotypes into four groups (Jombart 2008). LD decay was estimated using the software PopLDdecay v.3.41 (Zhang et al. 2019) using the parameter “-MaxDist 500”. Script Plot_OnePop.pl in the package was then used to plot the estimated *r*^2^ values over 10 kb bins. The *r*^2^ threshold was set to 0.2.

### GWAS analysis

Marker trait associations (MTAs) were calculated by combining the filtered SNP dataset of the genotypes with the corresponding BLUPs in R software using the Genome Association and Prediction Integrated Tool (GAPIT) version 2 package (Tang et al. 2016). The enriched Compressed Mixed Linear Model (ECMLM) method which builds on the Compressed/Mixed linear model factors by grouping individuals into clusters and stipulates the relationship among groups to correct for population structure (Li et al. 2014) was used as below;

$$y = X\beta + Zu + e$$

where ***y*** is a vector of the phenotype (disease levels); ***β*** represents unknown fixed effects, as well as population structure and marker effects; ***u*** is a vector of size s (number of groups) for unknown random polygenic effects following a distribution with mean of zero and covariance matrix of $G = 2K\sigma_{a}^{2}$ and *K* is the group kinship matrix with element $K_{{{\text{ij}}}} \left( {i, j = 1, 2,.... s} \right)$ representing the relationship between group *i* and *j*, and $\sigma_{a}^{2}$ is an unknown genetic variance. ***X*** and ***Z*** are matrices for ***β*** and ***u*** while ***e*** is a vector of random residual effects that are normally distributed with zero mean and covariance $R = I\sigma_{e}^{2}$. where *I* is the identity matrix and $\sigma_{e}^{2}$ is the unknown residual variance.

The resulting associations were displayed as Manhattan plots alongside quantile–quantile (Q–Q) plots to demonstrate the model fitness using qqman package in R (Turner 2018). The *P* values for each marker were adjusted for false discovery rate (FDR) (Benjamini and Hochberg 1995) and used to select significant associations (*P* < 0.05). Candidate genes were identified within 250 kbp distance of the significant marker using *Arachis duranensis* and *Arachis ipaensis* reference genomes. Information on the location of the genes and their annotations were obtained from the *A. ipaensis, A. duranensis* and annotation files (https//peanutbase.org/).

### Identification of haplotypes

Stable markers within identified significant QTL regions were used as references for building the haplotype blocks. All markers that were within the LD decay distance of 250 kbp made up a haplotype block. Individuals with ambiguous nucleotide calls were excluded from analysis. Phenotypic data were categorized based on the identified haplotypes and used to test for association. One way ANOVA with Duncan's test as a post hoc test was used to identify significant associations and measure specific differences between pairs of means in *R* using package DescTools ( et al. 2021). Only haplotypes that were present in at least five or more genotypes were considered for the statistical analysis. Further, Haploview v4.2 (Barrett et al. 2005) was used to visualize the presence of LD between the SNP markers within the significant haplotypes. We used combined dataset analysis to identify genotypes harboring unique haplotypes and further established the extent of diversity among the resistant genotypes in comparison to the African core collection.

## Results

### Phenotypic variation

**Fig. 2:**
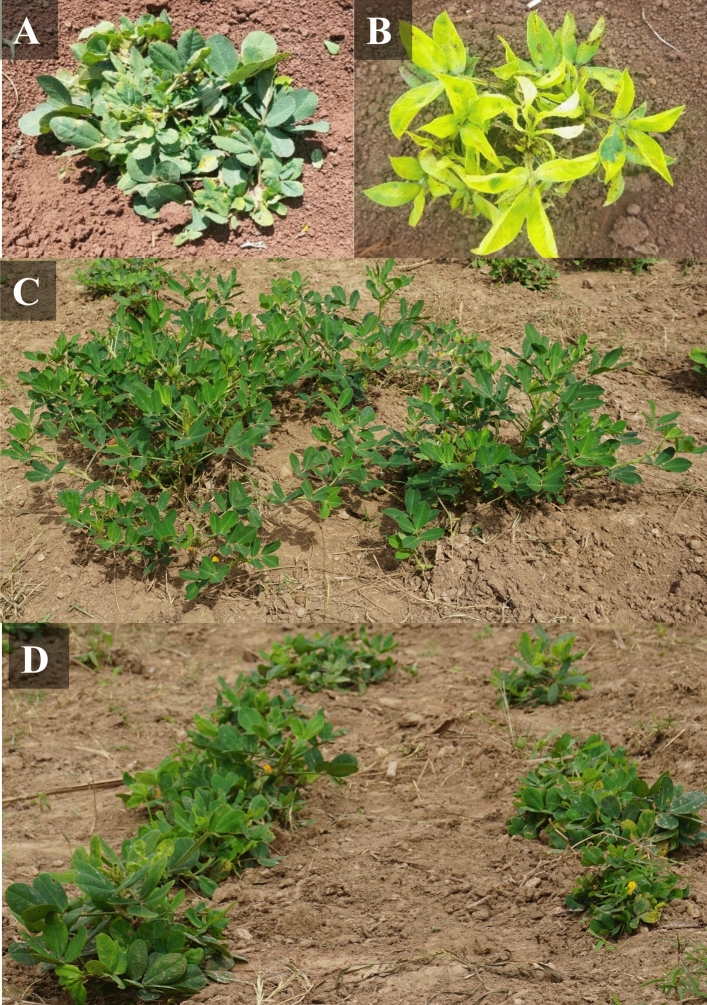
Symptoms of Groundnut Rosette Disease as observed in the field. **A**. Green rosette. **B**. Yellow rosette. **C**. Plot showing resistant check with 0% disease incidence at 60 DAP. **D**. Plot showing susceptible check with 100% PDI (all plants affected by GRD showing severe stuntedness) at 60 DAP

**Fig. 3:**
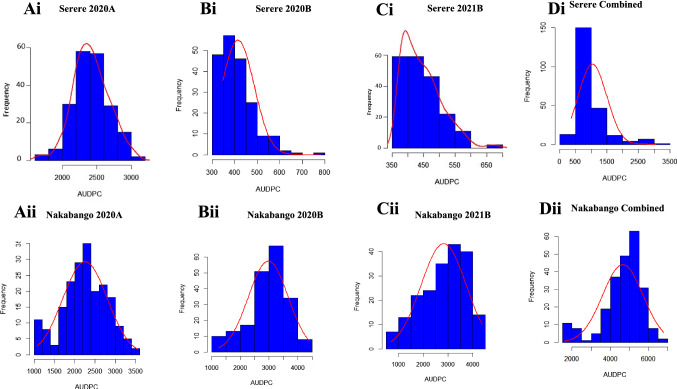
Phenotypic distribution of AUDPC across the two locations (Nakabango and Serere) for all seasons tested (2020A, 2020B, 2021B). The curves were drawn using BLUPs. There was no consistency in the distribution of the trait in Serere location (Ai, Bi, Ci and Di) as compared to Nakabango (Aii, Bii, Cii, Dii)

Table 1 provides descriptive statistics for the response of groundnut germplasm to GRD. The most common symptoms observed were green and yellow rosette (Fig. 2). Highly significant differences (*P* < 0.001) were observed among the genotypes for AUDPC across all the Nakabango trials (Table 1). At Serere, data revealed significant differences (*P* < 0.05) among the genotypes in seasons 2020A and 2020B. There were no significant differences observed for Serere 2021B. The broad sense heritability was low (0–30%) for environments Serere 2020B and Serere 2021B; moderate (31–60%) for environments Serere 2020A, Nakabango 2020A, 2020B and high (> 60%) for Nakabango 2021B. The frequency distribution graphs for AUDPC showed near normal distribution for environments Serere 2020A and Nakabango 2020A while for Serere 2020B and 2021B, AUDPC values were skewed to the right (Fig. 3). Environments Nakabango 2020B and 2021B were normally distributed (Fig. 3).

**Table 1:** Descriptive statistics for AUDPC across environments

Environment	2020A			2020B			2021B		
	Mean	MS	H^2^ (%)	Mean	MS	H^2^ (%)	Mean	MS	H^2^ (%)
Serere	2428.8	1,337,811**	31	404.7	420,363**	17	400.8	305288 ns	29
Nakabango	2261.7	2,339,754***	51	8643.6	22,515,350***	58	2819.6	3,624,008***	68

*MS* is the mean square value; *H*^2^—Broad sense heritability; **Significant at *P* < 0.005; ***Significant at *P* < 0.001; ns–non-significant

### Linkage disequilibrium, genetic diversity and population structure

**Fig. 4:**
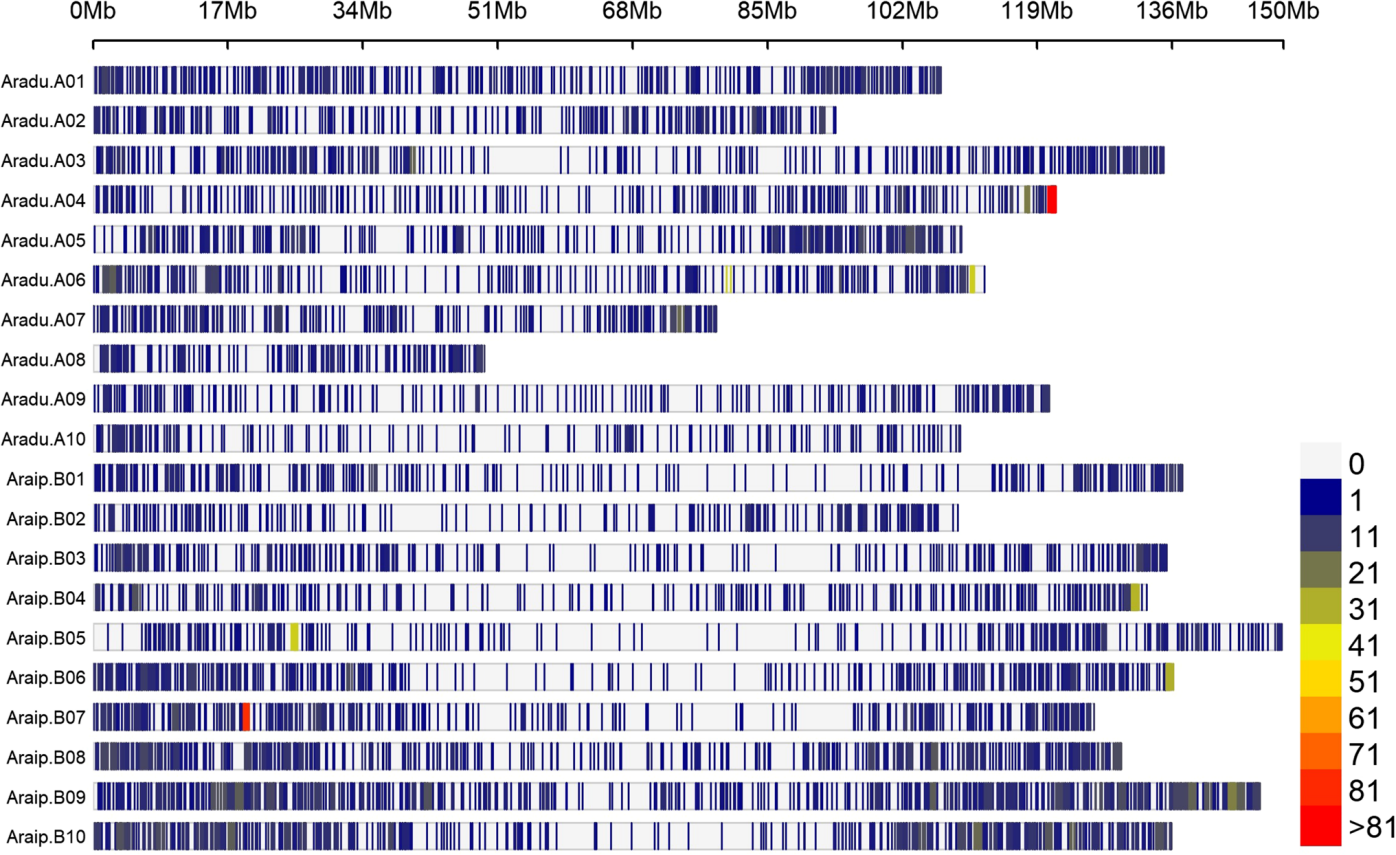
Distribution of high quality SNPs retained for population and marker-trait analysis against the joint *A. ipaensis* and *A. duranensis* reference genomes

A total of 7523 (3125 from sub-genome A and 4,398 from sub-genome B) high quality SNP markers were retained that had a genotype call rate > 0.95 and Minor Allele Frequency (MAF) > 0.05. The SNPs were well distributed across the 20 chromosomes (Fig. 4), with SNP densities of 2.5 and 2.8 SNPs/Mbp for sub-genomes A and B, respectively. The overall LD decay across the 20 chromosomes was estimated at 250 kbp (Supplementary Fig. 1). LD decayed more rapidly in the B sub-genome (177 kbp) in comparison to the A sub-genome (388 kbp) (Supplementary Fig. 1).

**Fig. 5:**
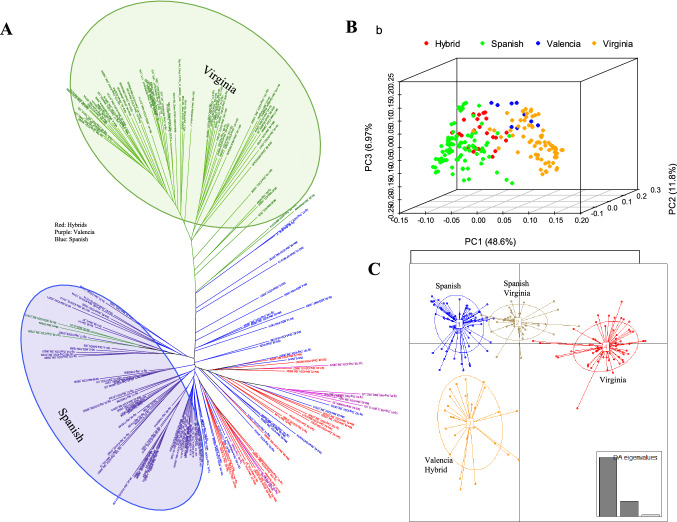
Relatedness of genotypes used in the study. **A**. A NJ tree revealing two major clusters comprising of Virginia and Spanish biological groups. Hybrid, Valencia and a number of Spanish genotypes appeared as admixtures. **B**. A PCA plot showing consistent clustering of the groundnut genotypes according to biological groups. The 3 PCs explained 67% genetic variation across the genotypes. **C**. A population structure analysis using DAPC that clustered the genotypes into 4 groups, of which the Spanish and Virginia clusters are the most distinct

The Neighbor-Joining dendrogram, PCA and DAPC all grouped the groundnut genotypes according to market class and not by country of origin (Fig. 5A–C). The Virginia and Spanish group clusters were the most distinct with minimal contamination within the major clusters (Fig. 5). Clusters 2 and 3 within the DAPC analysis were composed of a mixture of Spanish and Virginia (cluster 2), and Valencia and Hybrid (cluster 3) (Fig. 5C). Although a few Virginia genotypes clustered with the Spanish, there were no Spanish genotypes that clustered with the Virginia genotypes. The first 3 PCs explained a total of 67.3% (48.6%, 11.8% and 6.9%) genetic variation across the genotypes indicating the superior quality of SNPs used in the analysis (Fig. 5B).

### Genomic regions associated with GRD resistance

Due to low disease pressure in Serere that resulted in the lack of significant genetic variation in the response of genotypes to GRD for season 2021B, this dataset was not included in the GWAS analysis. Both the genotypic and phenotypic datasets that were used for GWAS have been made available at this link (https://figshare.com/s/ebf602b52ea2c5507f26). GWAS for Serere 2020A and 2020B yielded no significant markers (Supplementary Fig. 2) and for that reason, were not used for any further analysis or data interpretation. All the results presented below are for Nakabango.

**Fig. 6:**
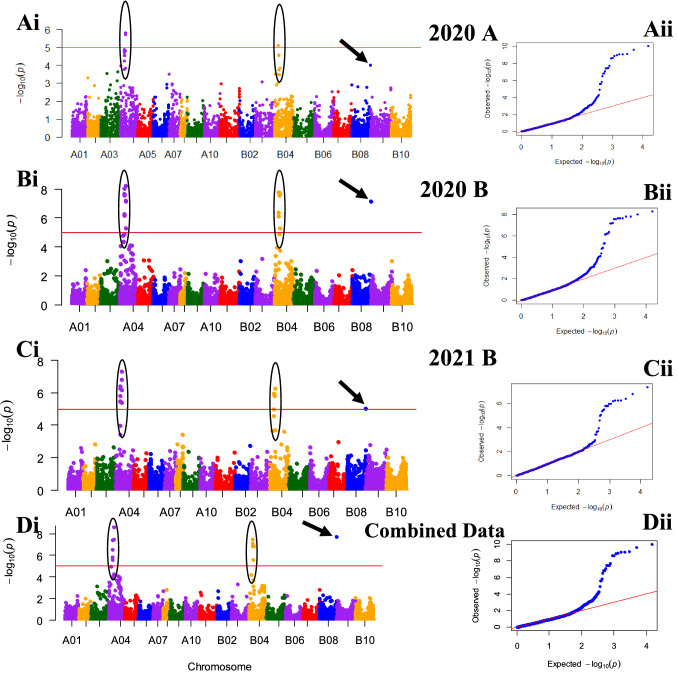
Manhattan (Ai, Bi, Ci and Di) and QQ (Aii, Bii, Cii, Dii) plots drawn using ECMLM approach indicating SNPs significantly associated with resistance to GRD for Nakabango. The consistent peaks on the Manhattan plots are highlighted on chromosomes A04 and B04. An additional signal on chromosome B08 is indicated by an arrow. The solid red line across the Manhattan plots represents the significance threshold based on FDR correction (*P* < 0.05). Manhattan (Ei, Fi and Gi) and QQ (Eii, Fii and Gii) plots show GWAS results for Serere. No SNPs were significant at FDR threshold of *P* < 0.05

Thirty-two significant marker-trait associations (MTAs) (FDR *P* < 0.05) were detected in at least one season and/or from combined seasons in Nakabango (Table 2; Supplementary Table 2). Manhattan and QQ plots supporting the GWAS results are provided in Fig. 6. Of the 32 markers detected, 21 were from chromosome A04 while 10 were from chromosome B04, which is syntenic to A04 (Fig. 6). One marker was detected on chromosome B08 (Table 2) and was supported with two seasons data as well as the combined dataset (Fig. 6). Eleven markers (AX-147219783, AX-147219785, AX-147219808, AX-147219820, AX-147219834, AX-147219906, AX-147219910, AX-147219925, AX-147247475, AX-147247493, AX-147247508) were common in all the four (2020A, 2020B, 2021B and combined) datasets (Table 2). The season 2020B reported the highest number of MTAs (30 SNPs) followed by the combined dataset with 26 MTAs (Table 2). The Percent Variation Explained (PVE) that was estimated based on *R*^2^ ranged from 0.25 to 0.29 (2020A), 0.34–0.42 (2020B), 0.32–0.38 (2021B) and 0.40–0.49 (combined dataset) (Supplementary Table 2). The combined dataset picked up an association with one additional SNP that was not detected with any of the single season’s data (AX-147219934).

**Table 2:** Marker-Trait Associations detected from each season and combined datasets and their respective *P* values

SNP	Chromosome	Position	*P* values			
			2020A	2020B	2021B	Combined datasets
1. AX-147219775	A04	27,460,853	N/A	4.21E-05	N/A	9.39E-05
2. AX-147219783	A04	27,962,232	5.74E-05	6.52E-07	1.02E-04	3.01E-07
3. AX-147219784	A04	27,963,468	1.36E-05	5.59E-07	N/A	1.51E-06
4. AX-147219785	A04	27,964,131	2.92E-05	2.20E-08	6.07E-07	N/A
5. AX-147219808	A04	29,244,932	1.93E-05	6.91E-07	2.26E-06	3.64E-08
6. AX-147219820	A04	30,792,252	1.52E-05	2.79E-08	4.15E-06	7.59E-10
7. AX-147219834	A04	31,201,852	1.48E-05	9.88E-09	3.48E-07	2.32E-10
8. AX-147219844	A04	31,447,199	N/A	1.31E-05	1.55E-06	2.59E-06
9. AX-147219906	A04	38,089,388	1.97E-06	4.79E-06	9.68E-08	3.38E-08
10. AX-147219910	A04	38,629,872	1.55E-05	7.01E-08	7.41E-07	2.19E-09
11. AX-147219924	A04	39,355,923	N/A	6.53E-08	5.22E-06	2.41E-09
12. AX-147219925	A04	39,358,203	1.52E-06	5.53E-09	6.51E-08	9.20E-11
13. AX-147219934	A04	86,907,162	N/A	N/A	N/A	5.19E-05
14. AX-147247470	B04	27,243,560	N/A	4.26E-07	7.42E-05	8.15E-08
15. AX-147247475	B04	27,988,550	8.02E-06	1.62E-08	1.14E-06	3.51E-08
16. AX-147247493	B04	29,672,339	2.86E-05	1.62E-08	1.14E-06	1.04E-09
17. AX-147247505	B04	30,758,471	N/A	1.31E-05	1.55E-06	2.59E-06
18. AX-147247508	B04	30,760,719	2.73E-05	2.70E-08	1.56E-06	1.25E-09
19. AX-147247536	B04	36,984,423	N/A	5.17E-06	N/A	1.34E-07
20. AX-147247549	B04	38,566,259	N/A	2.20E-08	6.07E-07	8.35E-10
21. AX-176791378	B04	25,635,381	N/A	7.31E-07	1.27E-05	1.47E-07
22. AX-176795814	A04	20,130,699	N/A	1.59E-05	N/A	1.17E-05
23. AX-176797377	B04	34,737,194	N/A	1.81E-04	N/A	8.36E-05
24. AX-176799050	A04	69,311,317	N/A	7.71E-05	N/A	5.99E-05
25. AX-176799431	A04	68,633,760	N/A	4.21E-05	N/A	9.74E-05
26. AX-176801644	B04	15,741,129	N/A	6.52E-07	N/A	8.35E-10
27. AX-176801951	A04	61,227,858	N/A	9.78E-05	N/A	1.90E-08
28. AX-176810563	A04	83,062,040	N/A	1.18E-04	N/A	N/A
29. AX-176814531	A04	4,507,325	N/A	8.68E-05	N/A	N/A
30. AX-176815600	A04	83,426,568	N/A	7.88E-05	N/A	N/A
31. AX-176823509	A04	32,085,166	2.83E-05	2.20E-08	6.07E-07	N/A
32. AX-177638322	B08	129,079,001	N/A	7.01E-08	1.12E-05	N/A

All markers shown were significant (FDR corrected *P* < 0.05)

### Haplotype-based association analysis

**Fig. 7:**
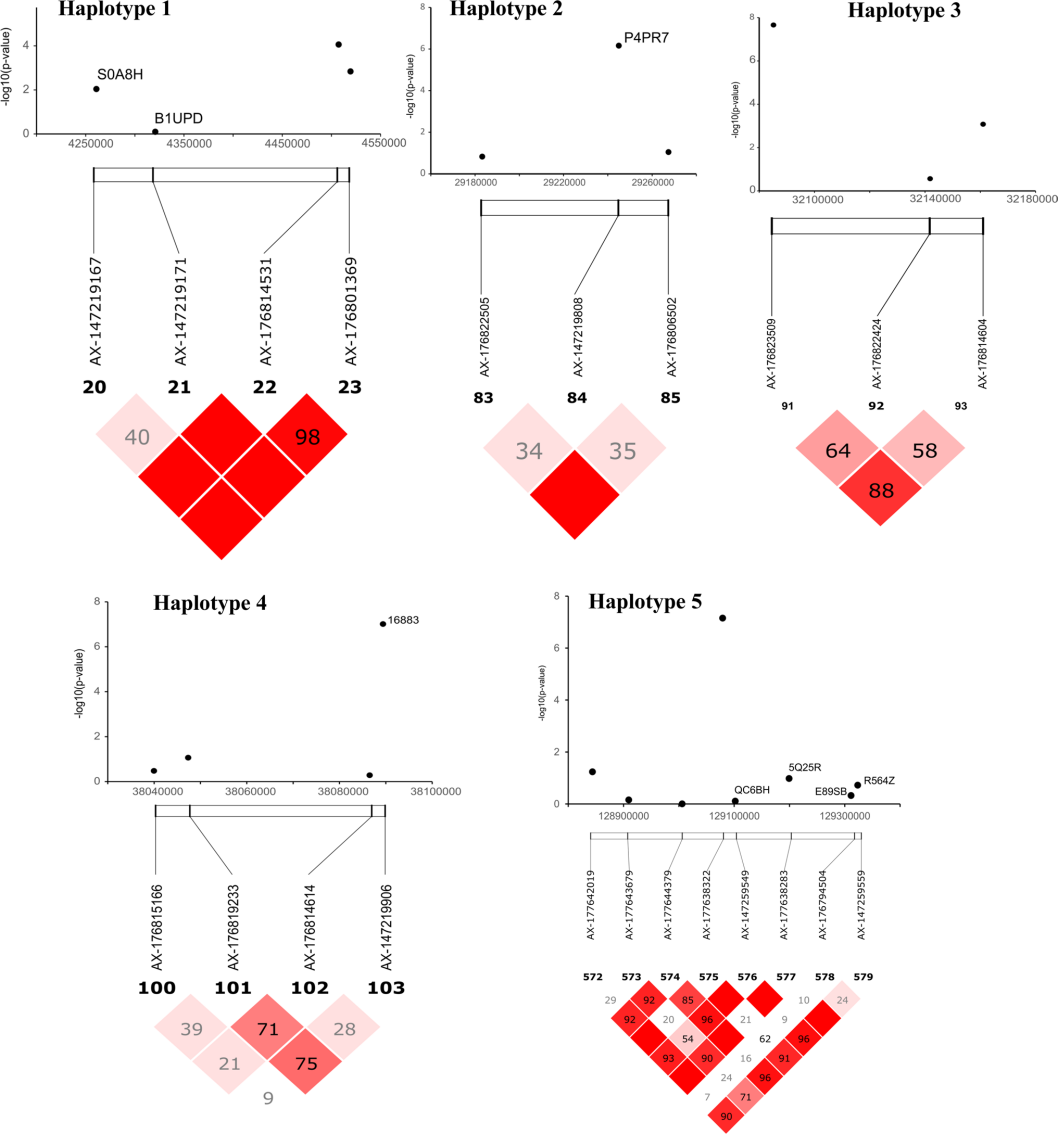
Five haplotypes significantly associated to GRD resistance. All the haplotypes were located in the QTL region of chromosome A04 except haplotype 5

We identified five haplotypes from the respective QTL regions that were associated with resistance to GRD (Table 3; Fig. 7). All the haplotypes were located on chromosome A04 except one that was located on chromosome B08. Tests of significance for all the possible allelic combinations at each haplotype block is given in Supplementary Table 3. Box plots drawn using each season and combined data confirmed the differences in performance between the favorable haplotypes and the alternative allelic combinations (Supplementary Fig. 3). One of the haplotype blocks (TGAA), was just 1 Mbp away from a major disease resistance gene (TIR-NBS-LRR) (Supplementary Table 4).

**Table 3:** Haplotypes associated with GRD resistance

Haplotype #	SNP Markers	Chromosome	Position (bp)	Number of genotypes harboring favorable haplotype
1. AAAA	AX-147219167	Aradu.A04	4,260,861	39 (18 from Uganda; 6 from Zambia; 5 from Ghana; 4 from Senegal, 3 from Malawi and 1 each from Togo, Mozambique and Mali)
	AX-147219171	Aradu.A04	4,320,760	
	AX-176814531	Aradu.A04	4,507,325	
	AX-176801369	Aradu.A04	4,519,432	
2. CCT	AX-176822505	Aradu.A04	29,183,214	25 (12 from Uganda; 3 each from Ghana, Mali and Senegal; 2 from Zambia and 1 each from Malawi and Togo)
	AX-147219808	Aradu.A04	29,244,932	
	AX-176806502	Aradu.A04	29,267,392	
3. CCA	AX-176823509	Aradu.A04	32,085,166	13 all from Uganda
	AX-176822424	Aradu.A04	32,141,842	
	AX-176814604	Aradu.A04	32,161,004	
4. TGAA	AX-176815166	Aradu.A04	38,039,961	9 (3 from Uganda, 2 each from Ghana and Zambia; 1 each from Malawi and Senegal)
	AX-176819233	Aradu.A04	38,047,338	
	AX-176814614	Aradu.A04	38,086,506	
	AX-147219906	Aradu.A04	38,089,388	
5. CTGTCGCA	AX-177642019	Araip.B08	128,843,602	9 (8 from Uganda and 1 from Malawi)
	AX-177643679	Araip.B08	128,909,268	
	AX-177644379	Araip.B08	129,005,860	
	AX-177638322	Araip.B08	129,079,001	
	AX-147259549	Araip.B08	129,102,004	
	AX-177638283	Araip.B08	129,199,206	
	AX-176794504	Araip.B08	129,311,474	
	AX-147259559	Araip.B08	129,323,492

**Fig. 8:**
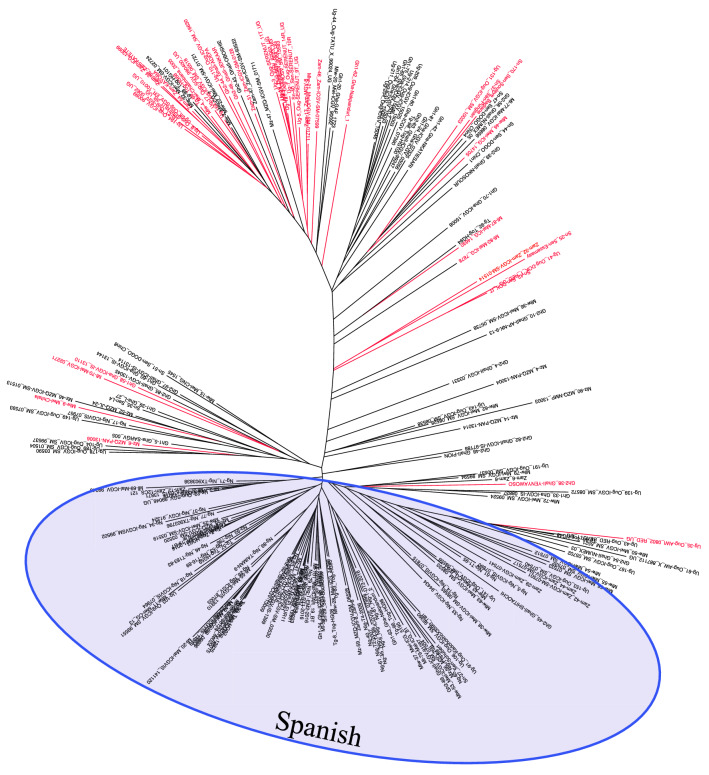
A N-J dendrogram showing the genetic diversity of the stable GRD resistant material in comparison with the African core set. The GRD resistant lines are highlighted in red. The predominantly Spanish cluster is highlighted in blue (color figure online)

Using combined datasets, we identified 39, 25, 13, 9 and 9 genotypes that harbored favorable haplotypes 1, 2, 3, 4 and 5, respectively (Table 3). There were 46 non-redundant genotypes from the combined dataset that harbored at least one favorable haplotype (Supplementary Fig. 4). Two genotypes (Ug-5_Oug-SERENUT_9T_UG and Ug-164_Oug-ICGV_SM_06518) harbored all the favorable haplotypes (Supplementary Fig. 4). Most of the 46 genotypes were Virginia (39) types with only 5 being Spanish and one each as Valencia and Hybrid types. A majority of the 46 genotypes were from Uganda (Table 3) and were genetically similar, forming a cluster within the predominantly Virginia market class group (Fig. 8). There were hardly any GRD resistant genotypes among the Spanish market class cluster (Fig. 8).

### Identification of candidate genes

**Fig. 9:**
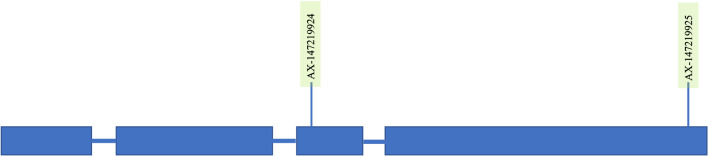
A sketch showing a hypothetical structure of the TIR-NBS-LRR disease resistance protein identified as a candidate gene on chromosome A04. Two markers co-localised on exons 3 and 4 are highlighted in light green. Figure not drawn to scale

We identified a non-redundant set of 383 genes within 250 kbp of all significant SNPs, of which 253 were from the A sub-genome (*Aradu*) while the remaining 130 genes were from the B sub-genome (*Araip*) (Supplementary Table 4). Of the 383 candidate genes identified, 62 (43 from sub-genome A and 19 from sub-genome B) were unknown proteins while an additional 37 (31 from sub-genome A and 6 from sub-genome B) were uncharacterized (Supplementary Table 4). A total of 17 markers were localized within genes, 10 on the A sub-genome and 7 on the B sub-genome (Supplementary Table 4). Two markers from sub-genome A, AX-147219924 and AX-147219925, were localized within a disease resistance protein (TIR-NBS-LRR; 39,354,055–39,358,311 bp) as shown in Fig. 9. There was a cluster of 9 “Disease resistance response proteins” on chromosome B04 that spanned from 15,5743, 372 bp to 15,709,800 bp. The other candidate genes onto which markers were localized included entatricopeptide (ppr) repeat-containing protein, peroxisome biogenesis protein 1-like isoform, protein root hair defective 3 homolog 2-like, vesicle-associated membrane protein 725, exocyst complex component 84b, Myosin heavy chain-related protein, Poly(rc)-binding protein 3-like protein, Ser/thr-rich protein t10 in dgcr region-like protein, argonaute family protein, Zip zinc/iron transport family protein and Phosphate transporter 1 (Supplementary Table 4).

### Stability of GRD resistance across seasons

Most of the top 20 most resistant groundnut genotypes showed stability across all the three seasons, with 14 genotypes showing top levels of resistance in all the three seasons, while an additional five were stable across two seasons (Table 4). Expectedly, Ugandan accessions dominated the top 20 performers category recording 9 stable genotypes across all seasons and another 3 genotypes across 2 seasons.

**Table 4:** A compilation of the most stable genotypes out of the top 20 best performers per season

Across all seasons	2020A + 2020B only	2020B + 2021B only
1. Ug-23_Oug-SGV_0084_UG	1. UGh2-46_GhaII-JENKAAR	1. Gh1-62_Gha-Nakpanduri_1
2. Ug-3_Oug-SERENUT_11T_UG	2. Mlw-46_Mwi-ICG_14705	2. Ug-7_Oug-SERENUT_14R_UG
3. Ug-121_Oug-ICGV_SM_15583	3. Ug-8_Oug-SERENUT_3R_UG	
4. Ug-19_Oug-SGV_07002_UG		
5. Ug-41_Oug-DOK_1_RED_UG		
6. Ug-164_Oug-ICGV_SM_06518		
7. Ug-5_Oug-SERENUT_9T_UG		
8. Ug-194_Oug-ICGV_90099		
9. Ug-28_Oug-SGV_ER_10010_UG		
10. Sn-40_Sen-SERENUT_10R		
11. Gh2-38_GhaII-YENYAWOSO		
12. Zam-17_Zam-MGV-8		
13. Zam-32_Zam-ICGV-SM-01514		
14. Mlw-21_Mwi-ICGV-SM_01711		

## Discussion

We sought to shed light on the genetic basis of Groundnut Rosette Disease (GRD) resistance using a diverse set of cultivated groundnut germplasm that had been carefully selected using genotypic data and breeders’ preferences. We identified significant Marker Trait Associations (MTAs), favourable haplotypes and candidate genes tightly linked to significant markers. Our key findings reveal important aspects on choice of germplasm, environments, molecular markers, the identification of candidate genes and their implications on future genetic and genomic studies for GRD resistance in groundnut.

### The choice of genotypes and environments

We have demonstrated that the African core collection was suitable for the identification of GRD resistance loci. We also captured the predominant groundnut market classes used across Africa. Collections with useful diversity such as core and mini core sets have been recommended as more appropriate for association studies as a result of numerous rounds of historical recombination (Otyama et al. 2019). Despite this careful selection of germplasm, our results also revealed the minimal existence of GRD resistant sources from the Spanish market class. Thirty-nine out of 46 (84.8%) of GRD resistant genotypes were from the Virginia class compared with 5 out of 46 (10.9%) from the Spanish class despite the Spanish class having the highest number of genotypes (97) in the evaluation set. This finding was inconsistent with earlier reports (Subrahmanyam et al. 1998) but may also have to do with the region where the experiment was undertaken and the existing pathogen isolates. The experiment was done in Uganda where majority of the breeding lines are Virginia types that have been bred for resistance to the pathogen isolates specific to the region. The Ugandan breeding lines were therefore more likely to be adapted to the pathogen isolates, and hence better performance. It is not surprising therefore that a majority of the GRD resistant and stable genotypes were from Uganda. Future studies will need to screen the same set of germplasm across different African ecologies to fully confirm their stability across different locations that might have different GRD pathogen isolates. Deom et al. (2000) reported region-specific clustering of GRV and sat-RNA isolates in comparison to GRAV, further indicating that a thorough characterisation of the GRD agents will be necessary for future gene and marker discovery studies.

Future studies will need to decipher what forms of satRNA are responsible for the different symptoms observed. While the current study reported mainly the yellow and green rosette, all the three major GRD symptoms have been previously reported in Eastern Africa, ranging from green (Okello et al. 2014, 2017; Mabele et al. 2021), chlorotic/yellow (Okello et al. 2017; Mabele et al. 2021) and mosaic rosette (Mukoye et al. 2020). It will be extremely important to partition the various symptoms and fully understand the corresponding agents or forms of satRNA responsible for the specific symptoms. The focus of this initial study was to determine broadly, resistance versus susceptibility under Ugandan GRD hotspots. The Area Under Disease Progress Curve (AUDPC) tool was useful in providing a quantitative summary of GRD intensity over time for each of the genotypes observed. No doubt, GRD will not be fully understood unless the specific agents and forms of satRNA are fully characterised with the corresponding reactions that they elicit from different genotypes.

The lack of consistent disease pressure in Serere resulted in no significant marker-trait associations. Accurate phenotypic datasets are critical for successful GWAS results (Gage et al. 2018). Earlier studies in GRD also reported significant and positive correlation between broad sense heritability (*H*^*2*^_bs_*)* and increased disease pressure (Van der Merwe et al. 1999). Our results are a strong indication that future GRD studies should include artificial inoculation to enhance disease pressure and its uniformity across trials. Lack of sufficient disease pressure would result in poor detection of causal alleles, especially those with minor effects (Davis et al. 1990; Zheng et al. 2018). Studies in other crops have also reported the need for enhanced disease pressure for accurate QTL identification (Gowda et al. 2018; Sitonik et al. 2019). While we were not able to detect any QTLs for Serere, it is also not clear if there could have been causal alleles with minor effects that we may have missed for Nakabango location too. Previous investigations in GRD enhanced disease pressure by growing plants in the glasshouse and inoculating with viruliferous aphids that had been reared on GRAV-infected groundnut (Naidu and Kimmins 2007) or using the field-based infector-row technique (Bock and Nigam 1988). Nevertheless, the consistency of QTLs detected in Nakabango across three seasons under natural inoculation will remain significant and a strong foundation for future studies in GRD.

### The suitability of the markers used in population structure analysis and GWAS

We used SNP markers based on the two diploid reference genomes of *A. duranensis* (sub-genome A) and *A. ipaensis* (sub-genome B) (Bertioli et al. 2016). Though fewer than expected, these SNP markers were fairly evenly distributed across the chromosomes, worked extremely well and were highly informative for establishing the population structure, and subsequently for the GWAS. There was a consistent absence of markers at the top of chromosomes A05 and B05 that we attributed to tetrasomic recombination, which is quite frequent in groundnut (Leal-Bertioli et al. 2015). The choice of the best markers for genotyping groundnut is always difficult given the ploidy and the large genome size (2.7 Gb). Although SNP markers called from reduced genome representation libraries (Gupta et al. 2015; Zhao et al. 2016; Han et al. 2018) or from transcriptome sequencing (Chopra et al. 2015) have been used previously in groundnut, they tend to result in homoeologous SNP calls (Zhao et al. 2016; Peng et al. 2020) unless the very expensive option of whole genome resequencing (WGRS) at high coverage is applied (Agarwal et al. 2018) to improve accuracy of calls. The SNP markers used in the current study had earlier been validated using the HAPLOSWEEP pipeline, which applies a haplotype-based method to retain allelic polymorphisms between genotypes (Clevenger et al. 2018). A recent study comparing different SNP development pipelines recommended the use of Axiom Arachis2 48 K SNP array followed by HAPLOSWEEP as the most accurate pipeline resulting in informative homozygous SNP calls (Peng et al. 2020).

According to Korte and Farlow (2013), the power of GWAS to detect significant MTAs is dependent on the phenotypic variation explained (PVE) by the SNPs. The minimum PVE recorded in our current study was 25%, which suggests that the SNP markers used were informative and sufficiently captured the existing phenotypic variation. However, the overall low density of the SNPs significantly reduced the power to identify relevant genomic regions with higher resolutions. Disease resistance is generally known to be controlled by both qualitative and quantitative genes (Jiquel et al. 2021). Although the GWAS results point to qualitative resistance based on the two major peaks identified, it will be difficult to rule out the involvement of quantitative resistance, which was also strongly supported by the frequency distribution graphs. While the QTLs identified in the current study will form an important basis for further understanding of the genetics of GRD resistance, future studies will need to include higher marker densities that would ensure that each causal genomic region is adequately captured. The lower numbers of SNPs in the current study may have reduced the power to identify rare variants, especially those with small effects (Gibson 2012).

Linkage disequilibrium (LD) is the non-random association between alleles at different loci in a breeding population and is the result of interplay of several factors including linkage, population structure, relatedness, selection and genetic drift (Flint-Garcia et al. 2003; Bush and Moore 2012). Understanding the LD pattern is crucial in genomic analysis as it determines the resolution and power of association analysis for a given population. Our study observed an overall LD decay of 250 kbp, suggesting that at least 10,384 markers would have been required to adequately scan the genome (2.7 Gb) for the population that we studied. Although our markers were slightly less, they were extremely informative across the population and provided consistent QTL peaks across seasons, especially for Nakabango location. The large LD blocks reported in our study are common in self-pollinating crops and are much smaller than those reported in other groundnut studies (Pandey et al. 2014; Otyama et al. 2019; Zhang et al. 2020; Zhou et al. 2021; Li et al. 2022). Future studies will need to use significantly more markers with a higher number of diverse genotypes to enhance the resolution of association analysis. Additional markers will also be needed for the A sub-genome where the markers were fewer and LD decay much slower than in the B sub-genome, consistent with earlier reports (Zhao et al. 2017).

### Genetics of GRD resistance, candidate gene identification and haplotype analysis

Moderate (51%, 58%) and high (68%) broad sense heritability (BSH) estimates reported for Nakabango location in our study was lower than in other studies that had fewer germplasm, but also with enhanced disease pressure either using artificial inoculation (Amoah et al. 2016) or infector-rows (Kayondo et al. 2014; Nalugo et al. 2016). Although our frequency distribution curves suggested that GRD resistance in groundnut is a quantitative trait, the clear peaks identified from GWAS indicate the likely involvement of a few major genes. In addition, two of the significant markers were located on a disease resistance gene (TIR-NBS-LRR) on chromosome A04, and one of the markers was located on an argonaute family protein on chromosome B04. We identified four significant haplotypes on chromosome A04. Preceding studies conducted via conventional breeding approaches proposed that GRD resistance is simply inherited and controlled by a single dominant gene (Olorunju et al. 1992; Athanas 2015) or two independent recessive genes (Nigam and Bock 1990; Olorunju et al. 1992). More studies will still be required to conclude the genetic control of GRD in groundnut.

Resistance (*R*) genes, which encode mostly nucleotide-binding site and leucine-rich repeat (NBS-LRR) proteins (Dangl and Jones 2001; Yuksel et al. 2005; Mchale et al. 2006) facilitate the ability of plants to fight pathogens through an antiviral mechanism known as Effector-triggered immunity (ETI). NBS-LRR proteins do this by recognizing effectors released by pathogens which result in activation of downstream signaling pathways consequently triggering plant defense reaction toward various pathogens (Bao et al. 2018; Li et al. 2017; Dubey and Kunal 2018). In the NBS-LRR cluster of proteins, the Toll/interleukin-1 receptor (TIR) associated with GRD resistance in this study is the most common and has been reported to play a role in the detection of *Avr* proteins such as in the tobacco mosaic virus (TMV) (Dubey and Kunal 2018), in *Pseudomonas syringae* in *Arabidopsis thaliana* (Kim et al. 2009) and in a downy mildew-resistant genotype in grapevine (*Vitis vinifera* L.) (Li et al. 2017).

Argonaute family proteins have been implicated in RNA interference (RNAi), a gene silencing mechanism deployed by plants to fight viral infections by hindering expression of genes during and post transcription (Muhammad et al. 2019). The involvement of argonaute proteins in the specific translational control of viral transcripts has been anticipated as an essential factor in resistance against viruses arbitrated by NBS-LRR proteins (Marone et al. 2013). Future studies will not only need to validate the QTLs identified in the current study using bi-parental mapping populations, but also characterise all the candidate genes within these QTLs. The functional markers identified in the current study will be developed into easy-to-use marker assays and validated for future routine genotyping and early generation selection for GRD resistance.

Haplotype analysis has been used in groundnut to distinguish botanical varieties (Zheng et al. 2022) and characterize different traits of interest (Wang et al. 2018; Liu et al. 2022; Zou et al. 2022) in past studies. The five favourable haplotypes identified in the current study provide an immediate resource for marker development and functional gene identification. Developing marker assays targeting candidate genes within the haplotype blocks will be a more precise approach for identifying putative functional markers for routine selection for GRD resistance but will still need to be validated using bi-parental populations. Haplotype based markers, once validated, will distinguish any new recombination blocks of interest on the chromosome that produce any favorable or unfavorable phenotypes (Bhat et al. 2021). The two genotypes (Ug-5_Oug-SERENUT_9T_UG and Ug-164_Oug-ICGV_SM_06518), which harboured all the favourable haplotypes will be useful, both as donor parents for introgressing GRD resistance, but also as resources for better understanding the genetics and evolution of GRD resistance alleles.

## Conclusion

Our results open a new chapter for GRD resistance studies and breeding in groundnut in Africa. Our findings, which include the identification of novel genomic regions, associated haplotype blocks and putative candidate genes that affect GRD resistance, will pave the way for marker assisted breeding for GRD. Bi-parental mapping populations and routine marker assays will need to be developed for validating the genomic regions identified for more efficient selection for GRD resistance in the future. Given the complexity of the disease, future studies should be planned more carefully to enable the full understanding of the genetics of resistance to the various agents as well as the vector. While single location experiments will enhance our understanding of the genetics of resistance to individual isolates, the search for more durable resistance in farmer-preferred varieties should be undertaken across several locations and seasons under high disease pressure. The current collaboration that involves several African countries will form a solid backbone for future successful characterisation of the host, the vector, as well as the various pathogen agents. Several advanced breeding tools including Next Generation Sequencing (NGS), Rapid Generation Advance (RGA), digital data capture, precision phenotyping, as well as gene editing should be deployed appropriately to speed up the varietal development process and enhance our understanding of this disease.

## Supplementary Information

Below is the link to the electronic supplementary material.

Supplementary file1 (DOCX 998 KB)

Supplementary file2 (XLSX 16 KB)

Supplementary file 3 Table 2: A summary of significant marker trait association for 2020A, 2020B, 2021B and combined analysis.

Supplementary file4 (XLS 33 KB)

Supplementary file5 (XLSX 31 KB)

## Declarations

### Availability of data and material

All data generated or analysed during this study are included in this published article.

### Code availability

N/A.

## References

[CR1] Ahmed B, Egwuma H, Idris MK (2021). Groundnut (*Arachis hypogaea*) pod and haulm production in the tropical legume project states Nigeria. Afr J Agr Res.

[CR2] Agarwal G, Clevenger J, Pandey MK (2018). High-density genetic map using whole-genome resequencing for fine mapping and candidate gene discovery for disease resistance in peanut. Plant Biotechnol.

[CR3] Alqudah AM, Sallam A, Stephen Baenziger P, Börner A (2020). GWAS: fast-forwarding gene identification and characterization in temperate cereals: lessons from barley–a review. J Adv Res.

[CR4] Amoah RA, Akromah R, Asibuo JY, Oppong A, Nyadanu D, Agyeman A, Bediako AK (2016). Genetic control of resistance to rosette virus disease in groundnut (*Arachis hypogaea * L.). J Plant Breed Crop Sci.

[CR5] Arya SS, Salve AR, Chauhan S (2016). Peanuts as functional food: a review. J Food Sci Technol.

[CR6] Athanas J (2015) Phenotypic and molecular characterization of recombinant inbred groundnut lines for resistance to groundnut rosette disease. Dissertation, Sokoine University of Agriculture, Morogoro

[CR7] Awata LA, Ifie BE, Danquah E, Jumbo MB, Suresh LM, Gowda M, Marchelo-Dragga PW, Olsen MS, Shorinola O, Yao NK, Boddupalli PM (2021). Introgression of maize lethal necrosis resistance quantitative trait loci into susceptible maize populations and validation of the resistance under field conditions in Naivasha. Kenya. Front Plant Sci.

[CR8] Bao D, Ganbaatar O, Cui X, Yu R, Bao W, Falk BW, Wuriyanghan H (2018). Down-regulation of genes coding for core RNAi components and disease resistance proteins via corresponding microRNAs might be correlated with successful Soybean mosaic virus infection in soybean. Mol Plant Pathol.

[CR9] Barrett JC, Fry B, Maller J, Daly MJ (2005). Haploview: analysis and visualization of LD and haplotype maps. Bioinform.

[CR10] Bates D, Mächler M, Bolker B, Walker S (2015). Fitting linear mixed-effects models using lme4. J Stat Softw.

[CR11] Benjamini Y, Hochberg Y (1995). Controlling the false discovery rate: a practical and powerful approach to mutliple testing. J R Stat Soc.

[CR12] Bertioli DJ, Leal-Bertioli S, Thomas Stalker (2016) The peanut genome: the history of the consortium and the structure of the genome of cultivated peanut and its diploid ancestors. In: Stalker HT, Wilson RF (eds) Peanuts, genetics, processing, and utilization, 1^st^ edn. Academic and AOCS Press, pp 147–161. 10.1016/B978-1-63067-038-2.00005-8

[CR13] Bertioli DJ, Jenkins J, Clevenger J (2019). The genome sequence of segmental allotetraploid peanut *Arachis hypogaea*. Nat Genet.

[CR14] Bhat JA, Yu D, Bohra A, Ganie SA, Varshney RK (2021). Features and applications of haplotypes in crop breeding. Commun Biol.

[CR15] Bock K, Murant A, Rajeshwari R (1990). The nature of the resistance in groundnut to rosette disease. Ann Appl Biol.

[CR16] Bock KR, Nigam SN (1988) Methodology of groundnut rosette resistance screening and vector-ecology studies in Malawi. Proceedings of the collaborative research on groundnut rosette virus disease: summary proceedings of the consultative group meeting. 8–10 March 1987, Lilongwe, pp 7–10

[CR17] Bradbury PJ, Zhang Z, Kroon DE, Casstevens TM, Ramdoss Y, Buckler ES (2007). TASSEL: software for association mapping of complex traits in diverse samples. Bioinform.

[CR18] Bush WS, Moore JH (2012) Genome-wide association studies. PLoS Comput Biol 8(12). 10.1371/journal.pcbi.1002822 10.1371/journal.pcbi.1002822 PMC3531285 23300413

[CR19] Chopra R, Burow G, Farmer A, Mudge J, Simpson CE, Wilkins TA, Baring MR, Puppala N, Chamberlin KD, Burow MD (2015). Next-generation transcriptome sequencing, SNP discovery and validation in four market classes of peanut *Arachis hypogaea* L. Mol Genet Genom.

[CR20] Clevenger JP, Korani W, Ozias-Akins P, Jackson S (2018). Haplotype-based genotyping in polyploids. Front Plant Sci.

[CR21] Dangl JL, Jones DG (2001). Plant pathogens and integrated defence responses to infection. Nat.

[CR22] Davis DW, Engelkes CA, Groth JV (1990). Erosion of resistance to common leaf rust in exotic-derived maize during selection for other traits. Phytopathol.

[CR23] De Beukelaer H, Davenport G, Fack V (2018). Core hunter 3: flexible core subset selection. BMC Bioinform.

[CR24] Deom CM, Naidu RA, Chiyembekeza AJ, Ntare BR, Subrahmanyam P (2000). Sequence diversity within the three agents of groundnut rosette disease. Phytopathol.

[CR25] Desmae H, Janila P, Okori P, Pandey MK, Motagi BN, Monyo E, Mponda O, Okello DK, Sako D, Echeckwu C, Oteng-Frimpong R (2019). Genetics, genomics and breeding of groundnut (*Arachis hypogaea* L). Plant Breed.

[CR26] Dubey N, Kunal S (2018) Role of NBS-LRR proteins in plant defense. In: Singh A, Singh IK (eds) Molecular aspects of plant-pathogen interaction. Springer Singapore Pte Ltd, pp 115–138. 10.1007/978-981-10-7371-7_5

[CR27] FAOSTAT (2019) https://www.fao.org/faostat/en/#data/QCL. Accessed 13th June 2021

[CR28] Flint-Garcia SA, Thornsberry JM, Edward-IV SB (2003). Structure of linkage disequilibrium in plants. Ann Rev Plant Biol.

[CR29] Gage JL, De-Leon N, Clayton MK (2018) Comparing genome-wide association study results from different measurements of an underlying phenotype. G3*:* Genes, Genomes, Genetics 8(11): 3715–3722. 10.1534/g3.118.200700 10.1534/g3.118.200700 PMC6222562 30262522

[CR30] Gawel N, Jarret R (1991). A modified CTAB DNA extraction procedure for *Musa* and *Ipomoea*. Plant Mol Biol Rep.

[CR31] Gibson G (2012). Rare and common variants: twenty arguments. Nat Rev Genet.

[CR32] Gowda M, Beyene Y, Makumbi D, Semagn K, Olsen MS, Bright JM, Das B, Mugo S, Suresh LM, Prasanna BM (2018) Discovery and validation of genomic regions associated with resistance to maize lethal necrosis in four biparental populations. Mol Breed 38(5). 10.1007/s11032-018-0829-7 10.1007/s11032-018-0829-7 PMC5945787 29773962

[CR33] Gupta SK, Baek J, Carrasquilla-Garcia N, Penmetsa RV (2015). Genome-wide polymorphism detection in peanut using next-generation restriction-site-associated DNA (RAD) sequencing. Mol Breed.

[CR34] Han S, Yuan M, Clevenger JP, Li C, Hagan A, Zhang X, Chen C, He G (2018) A SNP-based linkage map revealed QTLs for resistance to early and late leaf spot diseases in peanut (*Arachis hypogaea* L) Front Plant Sci. 10.3389/fpls.2018.01012 10.3389/fpls.2018.01012 PMC6048419 30042783

[CR35] Herselman L, Thwaites R, Kimmins FM, Courtois B, Van der Merwe PJA, Seal SE (2004). Identification and mapping of AFLP markers linked to peanut (*Arachis hypogaea* L.) resistance to the aphid vector of groundnut rosette disease. Theor Appl Genet.

[CR36] Janila P, Pandey MK, Shasidhar Y, Variath MT, Sriswathi M, Khera P, Manohar SS, Nagesh P, Vishwakarma MK, Mishra GP, Radhakrishnan T, Manivannan N, Dobariya KL, Vasanthi RP, Varshney RK (2016). Molecular breeding for introgression of fatty acid desaturase mutant alleles (ahFAD2A and ahFAD2B) enhances oil quality in high and low oil containing peanut genotypes. Plant Sci.

[CR37] Jiquel A, Gervais J, Geistodt-Kiener A, Delourme R, Gay EJ, Ollivier B, Fudal I, Faure S, Balesdent MH, Rouxel T (2021). A gene-for-gene interaction involving a ‘late’ effector contributes to quantitative resistance to the stem canker disease in *Brassica napus*. New Phytol.

[CR38] Jombart T (2008). Adegenet: a R package for the multivariate analysis of genetic markers. Bioinform.

[CR39] Kayondo SI, Rubaihayo PR, Ntare BR, Gibson P, Edema R, Ozimati A, Okello DK (2014). Genetics of resistance to groundnut rosette virus disease. Afr Crop Sci J.

[CR40] Kim SH, Kwon S, Saha D, Anyanwu NC, Gassmann W (2009). Resistance to the *Pseudomonas syringae* effector HopA1 is governed by the TIR-NBS-LRR protein RPS6 and is enhanced by mutations in SRFR1 1 [W][OA]. Plant Physiol.

[CR41] King JC, Blumberg J, Ingwersen L, Jenab M, Tucker CL (2008). Tree nuts and peanuts as components of a healthy diet. J Nutr.

[CR42] Korani W, Clevenger JP, Chu Y, Ozias-Akins P (2019). Machine learning as an effective method for identifying true single nucleotide polymorphisms in polyploid plants. The Plant Genome.

[CR43] Korte A, Farlow A (2013). The advantages and limitations of trait analysis with GWAS: a review. Plant Methods.

[CR44] Kurapati S, Kommineni R, Variath MT (2021). Localization and gene action studies for kernel iron and zinc concentration in groundnut (*Arachis hypogaea* L.). Euphytica.

[CR45] Leal-Bertioli S, Shirasawa K, Abernathy B, Moretzsohn M, Chavarro C, Clevenger J, Ozias-Akins P, Jackson S, Bertioli D (2015). Tetrasomic recombination is surprisingly frequent in allotetraploid *Arachis*. Genet.

[CR46] Li L, Cui S, Dang P (2022). GWAS and bulked segregant analysis reveal the loci controlling growth habit-related traits in cultivated peanut (*Arachis hypogaea* L.). BMC Genom.

[CR47] Li M, Liu X, Bradbury P, Yu J, Zhang YM, Todhunter RJ, Buckler ES, Zhang Z (2014). Enrichment of statistical power for genome-wide association studies. BMC Biol.

[CR48] Li X, Zhang YL, Yin L, Lu J (2017). Overexpression of pathogen-induced grapevine TIR-NB-LRR gene VaRGA1 enhances disease resistance and drought and salt tolerance in *Nicotiana benthamiana*. Protoplasma.

[CR49] Ligges U, Maechler M (2003) scatterplot3d-an R package for visualizing multivariate data. J Stat Softw. 8(11):1–20. 10.18637/jss.v008.i11

[CR50] Liu Y, Shao L, Zhou J, Li R, Pandey MK, Han Y, Cui F, Zhang J, Guo F, Chen J, Shan S (2022). Genomic insights into the genetic signatures of selection and seed trait loci in cultivated peanut. J Adv Res.

[CR51] Lynch RE (1990). Resistance in peanut to major arthropod pests. Fla Entomol.

[CR52] Marone D, Russo MA, Laidò G, Leonardis AM, Mastrangelo AM (2013). Plant nucleotide binding site–leucine-rich repeat (NBS-LRR) genes: active guardians in host defense responses. Int J Mol Sci.

[CR53] Mchale L, Tan X, Koehl P, Michelmore RW (2006). Plant NBS-LRR proteins : adaptable guards. Genome Biol.

[CR54] Mabele AS, Were HK, Ndong'a MF, Mukoye B (2021). Occurrence and genetic diversity of groundnut rosette assistor virus in western Kenya. Crop Prot.

[CR55] Mienie CS, Pretorius AE (2013). Application of marker-assisted selection for ahFAD2A and ahFAD2B genes governing the high-oleic acid trait in South African groundnut cultivars (*Arachis hypogaea* L.). Afr J Biotechnol.

[CR56] Minja EM, Van der Merwe PJA, Kimmins FM, Subrahmanyam P (1999). Screening groundnut breeding lines for resistance to aphids, *Aphid craccivora* Koch. Int Arachis Newsl.

[CR57] Mora-Escobedo R, Hernández-Luna P, Joaquín-Torres IC, Ortiz-Moreno A, Robles-Ramírez MD (2015). Physicochemical properties and fatty acid profile of eight peanut varieties grown in Mexico. CyTA-J Food.

[CR58] Mukoye B, Mabele AS, Ndonga MF, Mangeni BC, Were HK (2020). Distribution of groundnut rosette disease and sequence diversity of groundnut rosette virus associated satellite RNA (Sat-RNA) in Western Kenya. Int J Genet Mol Biol.

[CR59] Muhammad T, Zhang F, Zhang Y, Liang Y (2019). RNA interference : a natural immune system of plants to counteract biotic stressors. Cells.

[CR60] Murant AF, Kumar IK (1990). Different variants of the satellite RNA of groundnut rosette virus are responsible for the chlorotic and green forms of groundnut rosette disease. Ann Appl Biol.

[CR61] Nabuuma D, Nakimbugwe D, Byaruhanga YB, Saalia FK, Phillips RD, Chen J (2013). Formulation of a drinkable peanut-based therapeutic food for malnourished children using plant sources. Int J Food Sci Nutr.

[CR62] Naidu RA, Kimmins FM, Deom CM, Subrahmanyam P, Chiyembekeza AJ, Van der Merwe P (1999). Groundnut rosette: a virus affecting groundnut production in sub-saharan Africa. Plant Dis.

[CR63] Naidu RA, Kimmins FM (2007). The effect of groundnut rosette assistor virus on the agronomic performance of four groundnut (*Arachis hypogaea* L.) genotypes. J Phytopathol.

[CR64] Nalugo RC, Wambi W, Sebbuliba JM, Okello DK, Puppala N (2016). Gene effects for resistance to groundnut rossette disease in exotic valencia groundnuts. Afr Crop Sci J.

[CR65] Nigam SN, Bock KR (1990). Inheritance of resistance to groundnut rosette virus in groundnut (*Arachis hypogaea* L.). Ann Appl Biol.

[CR66] Nigam SN, Prasada RD, Bhatnagar-Mathur P, Sharma KK (2012). Genetic management of virus diseases in peanut. Plant Breed Rev.

[CR67] Okello DK, Biruma M, Deom CM (2010). Overview of groundnuts research in Uganda : past present and future. Afr J Biotechnol.

[CR68] Okello DK, Monyo E, Deom CM, Ininda J, Oloka HK (2013) Groundnuts production guide for Uganda: recommended practices for farmers. National Agricultural Research Organisation, Entebbe, pp 1

[CR69] Okello DK, Akello L, Tukamuhabwa P, Odong T, Ochwo-Ssemakula M, Adriko J, Deom CM (2014). Groundnut rosette disease symptoms types distribution and management of the disease in Uganda. Afr J Plant Sci.

[CR70] Okello DK, Ugen M, Tukamuhabwa P, Ochwo-Ssemakula M, Odong T, Adriko J, Kiconco F, Male A, Deom CM (2017). Molecular diagnostics of groundnut rosette disease agents in Uganda : implications on epidemiology and management of groundnut rosette disease. J Plant Breed Crop Sci.

[CR71] Olorunju P (1992). Inheritance of resistance in peanut to mixed infections of groundnut rosette virus (GRV) and groundnut rosette assistor virus and a single infection of GRV. Plant Dis.

[CR72] Otyama P, Chamberlin K, Ozias-Akins P, Graham M, Cannon E, Cannon S, MacDonald G, Anglin N (2022) Genome-wide approaches delineate the additive, epistatic, and pleiotropic nature of variants controlling fatty acid composition in peanut (*Arachis hypogaea* L.). G3: Genes, Genomes, Genetics 12(1). 10.1093/G3JOURNAL/JKAB382 10.1093/g3journal/jkab382 PMC8728033 34751378

[CR73] Otyama PI, Wilkey A, Kulkarni R, Assefa T, Chu Y, Clevenger J, O’Connor DJ, Wright GC, Dezern SW, MacDonald GE, Anglin NL, Cannon EKS, Ozias-Akins P, Cannon SB (2019) Evaluation of linkage disequilibrium population structure and genetic diversity in the U.S. peanut mini core collection. BMC Genom 20(1):1–17. 10.1186/s12864-019-5824-9 10.1186/s12864-019-5824-9 PMC6558826 31185892

[CR74] Pandey MK, Upadhyaya HD, Rathore A et al (2014) Genomewide association studies for 50 agronomic traits in peanut using the ‘ reference set ’ comprising 300 genotypes from 48 countries of the semi-arid tropics of the world. PLoS ONE 9(8). 10.1371/journal.pone.0105228 10.1371/journal.pone.0105228 PMC4139351 25140620

[CR75] Pandey MK, Agarwal G, Sandip KM (2017). Development and evaluation of a high density genotyping “axiom-arachis” array with 58 k SNPs for accelerating genetics and breeding in groundnut. Sci Rep.

[CR76] Peng Z, Zhao Z, Clevenger JP, Chu Y, Paudel D, Ozias-Akins P and Wang J (2020) Comparison of SNP calling pipelines and NGS platforms to predict the genomic regions harboring candidate genes for nodulation in cultivated peanut. Front Genet 11:222. 10.3389/fgene.2020.00222 10.3389/fgene.2020.00222 PMC7105825 32265983

[CR77] R Core Team (2021). R: a language and environment for statistical computing. R foundation for statistical computing, Vienna, Austria. URL https://www.R-project.org

[CR78] Schoonees A, Lombard MJ, Musekiwa A, Nel E, Volmink J (2019) Ready-to-use therapeutic food (RUTF) for home-based nutritional rehabilitation of severe acute malnutrition in children from six months to five years of age. Cochrane Database of Systematic Reviews 2019(5). 10.1002/14651858.CD009000.pub3 10.1002/14651858.CD009000.pub3 PMC6537457 31090070

[CR79] Simko I, Piepho H (2012). The area under the disease progress stairs : calculation, advantage and application. Anal Theor Plant Pathol.

[CR80] Sitonik C, Suresh LM, Beyene Y, Olsen MS, Makumbi D, Oliver K, Das B, Bright JM, Mugo S, Crossa J, Tarekegne A, Prasanna BM, Gowda M (2019). Genetic architecture of maize chlorotic mottle virus and maize lethal necrosis through GWAS, linkage analysis and genomic prediction in tropical maize germplasm. Theor Appl Genet.

[CR81] Signorell A, Aho K, Alfons A et al (2021) DescTools: Tools for descriptive statistics. https://cran.r-project.org/package=DescTools. Accessed 13th January 2022

[CR82] Subrahmanyam P, Hildebrand GL, Naidu RA, Reddy LJ, Singh AK (1998). Sources of resistance to groundnut rosette disease in global groundnut germplasm. Ann Appl Biol.

[CR83] Taliansky ME, Robinson DJ, Murant AF (2000). Groundnut rosette disease virus complex: biology and molecular biology. Adv Virus Res.

[CR84] Tang Y, Liu X, Wang J, Li M, Wang Q, Tian F, Su Z, Pan Y, Liu D, Lipka AE, Buckler ES, Zhang Z (2016) GAPIT version 2: an enhanced integrated tool for genomic association and prediction. The Plant Genome 9(2). 10.3835/plantgenome2015.11.0120 10.3835/plantgenome2015.11.0120 27898829

[CR85] Toomer OT (2017). Nutritional chemistry of the peanut (*Arachis hypogaea*). Crit Rev Food Sci Nutr.

[CR86] Turner SD (2018). qqman: an R package for visualizing GWAS results using Q-Q and manhattan plots. J Open Source Softw.

[CR87] Usman A, Ofori K, Danquah EY, Offei SK, Ado SG (2015). Genetic analysis of groundnut rosette virus disease in groundnut (*Arachis hypogaea* L.). Afr J Plant Sci.

[CR88] Van der Merwe PA, Reddy L, Subrahmanyam JP, Naidu RA (1999). Criteria for selecting groundnut varieties in breeding for resistance to rosette disease. S Afr J Plant Soil.

[CR89] Wagh DD, Deore BR (2015). Ready to use therapeutic food (RUTF): an overview. Adv Life Sci Health.

[CR90] Waliyar F, Kumar PL, Ntare B, Monyo E, Nigam S, Reddy A, Osiru M, Diallo A (2007) A century of research on groundnut rosette disease and its management. Int Crops Res Inst Semi-Arid Tropics information bulletin 75

[CR91] Wang J, Yan C, Li Y, Li C, Zhao X, Yuan C, Sun Q, Shan S (2019). GWAS discovery of candidate genes for yield-related traits in peanut and support from earlier QTL mapping studies. Genes.

[CR92] Yin L, Zhang H, Tang Z, Xu J, Yin D, Zhang Z, Yuan X, Zhu M, Zhao S, Li X, Liu X (2021). rMVP: a memory-efficient, visualization-enhanced, and parallel-accelerated tool for genome-wide association study. Genom Proteom Bioinform.

[CR93] Wang X, Xu P, Yin L, Ren Y, Li S, Shi Y, Alcock TD, Xiong Q, Qian W, Chi X, Pandey MK (2018). Genomic and transcriptomic analysis identified gene clusters and candidate genes for oil content in peanut (*Arachis hypogaea* L.). Plant Mol Biol Report.

[CR94] Yuksel B, Estill JC, Schulze SR, Paterson AH (2005). Organization and evolution of resistance gene analogs in peanut. Mol Genet Genom.

[CR95] Zhang C, Dong SS, Xu JY, He WM, Yang TL (2019). PopLDdecay: a fast and effective tool for linkage disequilibrium decay analysis based on variant call format files. Bioinform.

[CR96] Zhang H, Chu Y, Dang P, Tang Y, Jiang T, Clevenger JP, Ozias-Akins P, Holbrook C, Wang ML, Campbell H, Hagan A, Chen C (2020). Identification of QTLs for resistance to leaf spots in cultivated peanut (*Arachis hypogaea* L.) through GWAS analysis. Theor Appl Genet.

[CR97] Zhang H, Wang ML, Dang P, Jiang T, Zhao S, Lamb M, Chen C (2021). Identification of potential QTLs and genes associated with seed composition traits in peanut (*Arachis hypogaea * L.) using GWAS and RNA-Seq analysis. Gene.

[CR98] Zhao Y, Zhang C, Chen H, Yuan M, Nipper R, Prakash CS, Zhuang W, He G (2016). QTL mapping for bacterial wilt resistance in peanut (*Arachis hypogaea* L.). Mol Breed.

[CR99] Zhao J, Huang L, Ren X, Pandey MK, Wu B, Chen Y, Zhou X, Chen W, Xia Y, Li Z, Luo H, Lei Y, Varshney RK, Liao B, Jiang H (2017). Genetic variation and association mapping of seed-related traits in cultivated peanut (*Arachis hypogaea* L.) using single-locus simple sequence repeat markers. Front Plant Sci.

[CR100] Zheng H, Chen J, Mu C, Makumbi D, Xu X, Mahuku G (2018). Combined linkage and association mapping reveal QTL for host plant resistance to common rust (*Puccinia sorghi*) in tropical maize. BMC Plant Biol.

[CR101] Zheng Z, Sun Z, Qi F, Fang Y, Lin K, Pavan S, Huang B, Dong W, Du P, Tian M, Shi L (2022) DNA sequencing sheds light on the evolutionary history of peanut and identifies genes associated with phenotypic diversification. 10.21203/rs.3.rs-1776558/v1

[CR102] Zhou X, Guo J, Pandey MK, Varshney RK, Huang L, Luo H, Liu N, Chen W, Lei Y, Liao B, Jiang H (2021). Dissection of the genetic basis of yield-related traits in the chinese peanut mini-core collection through genome-wide association studies. Front Plant Sci.

[CR103] Zou K, Kim KS, Kang D, Kim MC, Ha J, Moon JK, Jun TH (2022). Genome-wide association study of leaf chlorophyll content using high-density SNP array in peanuts (*Arachis hypogaea* L). Agron.

