# Supplementary material for: Genome-wide association studies reveal novel loci for resistance to groundnut rosette disease in the African core groundnut collection
Source: Theor Appl Genet. 2023 Mar 10;136(3):35. doi: 10.1007/s00122-023-04259-4 (PMC10006280; doi:10.1007/s00122-023-04259-4)
Supplement: Supplementary file 1 — Supplementary file1 (DOCX 998 KB) [file 122_2023_4259_MOESM1_ESM.docx]

**Supplementary Figures**

**Supplementary Figure 1** LD decay plots showing differences in the LD decay distance. **A.** An overall LD decay distance of 250 kbp. **B.** LD decay distance of 388 kbp for sub-genome A. **C.** LD decay distance of 177 kbp for sub-genome B

**Supplementary Figure 2** Manhattan and QQ plots generated after marker-trait-association (MTA) using BLUPs from phenotypic data collected from Serere. No obvious peaks were detected. **Ai.** Manhattan plot generated using Serere 2020A data. **Aii**. Corresponding QQ plot generated using observed data for 2020A versus expected. **Bi.** Manhattan plot generated using Serere 2020B data. **Bii.** Corresponding QQ plot generated using observed data for 2020B versus the expected. **Ci.** Manhattan plot generated using combined dataset from Serere 2020A and 2020B datasets. **Cii.** Corresponding QQ plot generated by plotting observed means for 2020A and 2020B versus the expected

**Supplementary Figure 3** Box plots drawn for the identified haplotypes showing clear differences between the favorable haplotypes (AAAA, CCT, CCA and CTGTCGCA) and alternative haplotypes across the different seasons tested


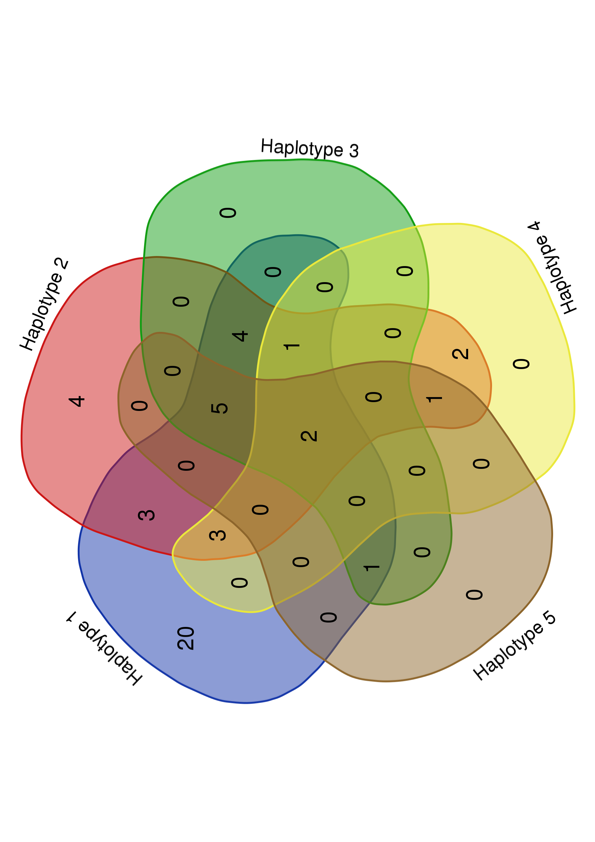


**Supplementary Figure 4**. A Venn diagram showing the number of genotypes harboring different haplotypes, and how the haplotypes are shared among them
